# Atf7ip Inhibits Osteoblast Differentiation via Negative Regulation of the Sp7 Transcription Factor

**DOI:** 10.3390/ijms24054305

**Published:** 2023-02-21

**Authors:** Guoqin Hu, Xian Shi, Xiuxia Qu, Chunqing Han, Anran Hu, Zhongtang Jia, Jiatao Yang, Huanliang Liu, Yu Wu

**Affiliations:** 1 Lab of Modern Environmental Toxicology, Department of Public Health and Preventive Medicine, Wuxi School of Medicine, Jiangnan University, Wuxi 214122, China; 2 Department of Basic Medicine, Wuxi School of Medicine, Jiangnan University, Wuxi 214122, China; 3 Environment and Health Research Division, Public Health Research Centre, Jiangnan University, Wuxi 214122, China

**Keywords:** Atf7ip, Setdb1, osteoblast differentiation, Sp7

## Abstract

Epigenetic modifications are critical for cell differentiation and growth. As a regulator of H3K9 methylation, Setdb1 is implicated in osteoblast proliferation and differentiation. The activity and nucleus localization of Setdb1 are regulated by its binding partner, Atf7ip. However, whether Atf7ip is involved in the regulation of osteoblast differentiation remains largely unclear. In the present study, we found that Atf7ip expression was upregulated during the osteogenesis of primary bone marrow stromal cells and MC3T3-E1 cells, and was induced in PTH-treated cells. The overexpression of Atf7ip impaired osteoblast differentiation in MC3T3-E1 cells regardless of PTH treatment, as measured by the expression of osteoblast differentiation markers, Alp-positive cells, Alp activity, and calcium deposition. Conversely, the depletion of Atf7ip in MC3T3-E1 cells promoted osteoblast differentiation. Compared with the control mice, animals with Atf7ip deletion in the osteoblasts (*Oc-Cre;Atf7ip^f/f^*) showed more bone formation and a significant increase in the bone trabeculae microarchitecture, as reflected by μ-CT and bone histomorphometry. Mechanistically, Atf7ip contributed to the nucleus localization of Setdb1 in MC3T3-E1, but did not affect Setdb1 expression. Atf7ip negatively regulated Sp7 expression, and through specific siRNA, Sp7 knockdown attenuated the enhancing role of Atf7ip deletion in osteoblast differentiation. Through these data, we identified Atf7ip as a novel negative regulator of osteogenesis, possibly via its epigenetic regulation of Sp7 expression, and demonstrated that Atf7ip inhibition is a potential therapeutic measure for enhancing bone formation.

## 1. Introduction

The formation of mature osteoblasts is regulated by specific transcription factors [1] (e.g., Runx2, Sp7 (Osterix)), the expressions of which are maintained at low levels in uncommitted mesenchymal multipotent cells and are stimulated during osteogenesis [2,3]. Multiple mechanisms are involved in this process, such as the synergized nuclear factors regulated by upstream developmental signaling [4]. Recently, proteins that govern chromatin structures were also found to be involved in the modulation of Runx2 and Sp7 expression [5]. To enable the expression of lineage-specific genes and the formation of terminally differentiated cells, the chromatin must maintain an active status in cell differentiation, which is largely controlled by DNA methylation and histone post-translational modifications (HPTMs) [5]. DNA methylation is often associated with gene silencing, but HPTMs can act as mediators of either gene activation or repression. HPTMs, such as the demethylation and acetylation of H3K27 and H3K9, are related to gene activation, whereas the methylation of H3K27 and H3K9 causes gene repression.

The methylation of H3K9, mediated by the lysine methyltransferases (KMTs) Suv39H1/H2, G9a, or Setdb1, contributes to the formation of highly compacted and transcriptionally repressed chromatin (heterochromatin) [5,6]. Setdb1, also known as ESET, is widely expressed in multiple cells and tissues and methylates histone H3, particularly at H3K9 [7]. The conserved N-terminal Tudor domain on Setdb1 aids in the connection with other chromatin-modifying enzymes, and the C-terminal SET zone catalyzes H3K9 methylation [8]. The specific knockout of Setdb1 in mesenchymal cells significantly damages osteoblast differentiation, which is related to the deregulation of Runx2 and Indian hedgehog (Ihh) [9]. Setdb1 also binds to the Sp7 promoter of C3H uncommitted multipotent cells, and this binding is diminished following osteogenic differentiation [2]. Moreover, the enzymatic activity of Setdb1 is modulated by a given binding partner, such as Atf7ip [6]. Atf7ip, also called as mAM or MCAF1, facilitates the conversion of H3K9 dimethyl to trimethyl by Setdb1 [10]. The complex formation of Atf7ip, MBD1, and Setdb1 is required for transcriptional repression, heterochromatin formation, and X chromosome inactivation, mediated by Setdb1 and MBD1 [11,12]. Atf7ip regulates Setdb1 catalytic activity, mainly via its regulation of the subcellular localization and stability of Setdb1 [13,14,15]. However, whether or not Atf7ip affects Runx2 and Sp7 expression so as to regulate osteoblast differentiation is unknown.

In this study, we ascertained the involvement of Atf7ip in osteoblast differentiation both in vitro and in vivo. This work not only reveals the negative role of Atf7ip in osteoblast differentiation, but also implicates the epigenetic mechanisms of Atf7ip in the regulation of osteogenesis via its regulation of Sp7.

## 2. Results

### 2.1. Atf7ip Expression Increases during Osteoblast Differentiation

Atf7ip is abundantly expressed in bone and bone marrow [16] and is implicated in the regulation of osteoblast proliferation [17]. To determine the expression pattern of Atf7ip in relation to osteoblastic differentiation, we measured its expression in pre-osteoblast MC3T3-E1 cells treated with osteoblast differentiation medium. Atf7ip expression was elevated, as were the osteoblast differentiation markers Runx2, Alp, Osteocalcin, and Col1a1 (Figure S1A–E). However, the expression of Setdb1, a binding partner of Atf7ip, was unchanged (Figure S1F).

**Figure 1:**
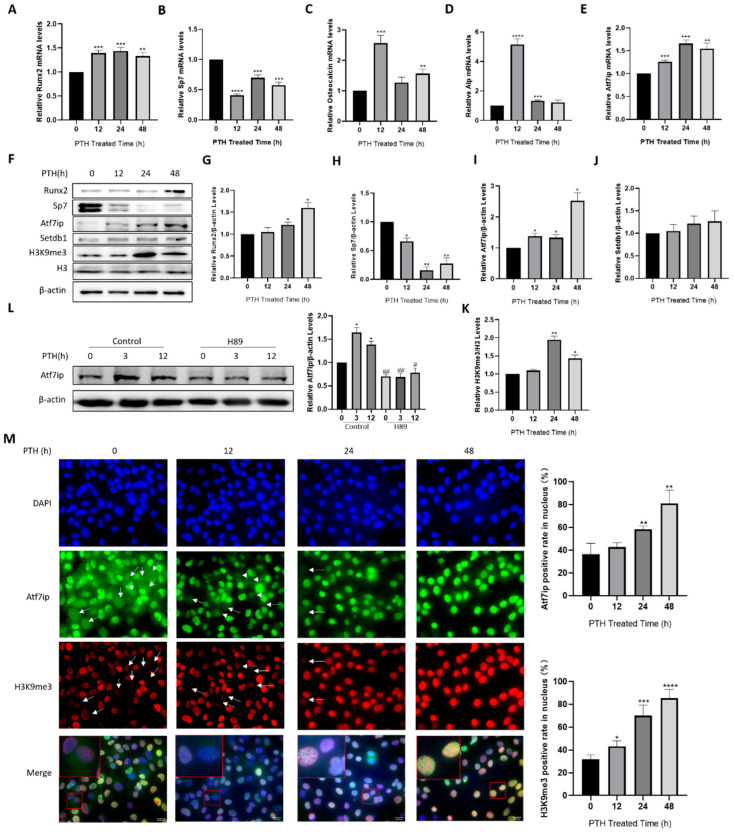
PTH induces Atf7ip expression in osteoblasts. MC3T3-E1 cells were treated with 500 ng/mL PTH for the indicated time. (**A**–**E**) The expressions of Runx2, Sp7, Osteocalcin, Alp, and Atf7ip were examined by real-time PCR, *n* = 3. (**F**–**K**) The protein expressions of Runx2, Sp7, Atf7ip, Setdb1, H3K9me3, and H3 were analyzed by Western blot, *n* = 3. (**L**) MC3T3-E1 cells were pretreated with 10 μM of H89 for 12 h and cultured for 12 h in medium with 500 ng/mL PTH. Atf7ip expression was examined by Western blot. β-actin was used as the loading control. Representative images from independent experiments are shown. The densitometric results were measured with ImageJ (the same below), *n* = 3. (**M**) MC3T3-E1 cells were treated with 500 ng/mL PTH for the indicated time. The subcellular localization of Atf7ip and H3K9me3 was determined by immunofluorescence. The arrows indicate the cells without nucleus localization of Atf7ip and H3K9me3, and the cells with nuclear localization of Atf7ip or H3K9me3 were calculated with Image J, *n* = 4. Scale bar, 20 μm. Selected sections magnified 10-fold. Data are represented as mean ± SD, * *p* < 0.05, ** *p* < 0.01, *** *p* < 0.001, **** *p* < 0.0001, treatments vs. no treatment. ^#^ *p* < 0.05, ^##^ *p* < 0.01, H89 vs. control, examined by one-way ANOVA.

To further demonstrate the expression pattern of Atf7ip during osteoblast differentiation, we adopted the model of osteoblast differentiation by the parathyroid hormone (PTH), an anabolic factor in bone formation, which promotes osteoblast differentiation and osteoblastic lineage commitment [18]. PTH induced the mRNA levels of Runx2, Osteocalcin, and Alp and elevated Runx2 protein expression, but inhibited Sp7 mRNA and protein expression (Figure 1A–K), as reported previously [19]. Using this model, we found that both the mRNA and protein levels of Atf7ip expression increased in a time-dependent manner in pre-osteoblast MC3T3-E1 cells (Figure 1A–K). Meanwhile, H3K9 tri-methylation (H3K9me3) was also elevated, although the Setdb1 protein level was unchanged (Figure 1A–K). In C3H/10T1/2 cells, the PTH treatment also increased Atf7ip expression and H3K9me3 in a time-dependent manner (Figure S2A–D). PTH interacts with its receptor PTH1R and activates PKA- or PKC-dependent signaling [18]. The cells pretreated with the PKA inhibitor H-89, but not the PKC inhibitor Sotrastaurin, inhibited the effect of PTH on Atf7ip expression in the MC3T3-E1 cells (Figure 1L and Figure S2E).

As the cellular localization of Atf7ip is important for its function and ability to bind and stabilize Setdb1 [15], we further verified the nature of Atf7ip localization in MC3T3-E1 cells and explored whether it was regulated by PTH. The immunofluorescence results showed that the ratio of cells with Atf7ip localized to nucleus in the control MC3T3-E1 cells was approximately 37%, which increased to 59% and 80% after 24 and 48 h of PTH treatment, respectively (Figure 1M). The number of cells that underwent H3K9me3 modification also consistently increased (Figure 1M). To ascertain whether this increased nucleus localization was due to increased protein expression upon PTH treatment, we pretreated the cells with CHX to interrupt the process of new protein generation. The results showed that the CHX treatment decreased Atf7ip nucleus localization in the PTH-treated cells (Hu.G., Wu.Y., Jiangnan University, Wuxi, China. The localization of Atf7ip in osteoblast, 2022). In summary, these results suggest a potential role of Atf7ip in regulating the osteogenic differentiation of osteoblasts.

### 2.2. Silencing of Atf7ip Expression Promotes MC3T3-E1 Osteogenesis

**Figure 2:**
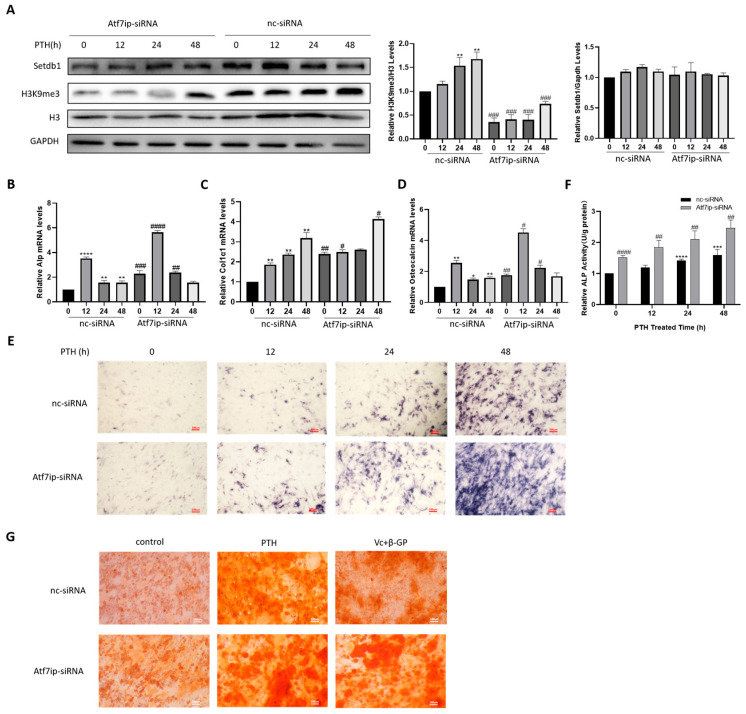
Silencing of Atf7ip expression promotes MC3T3-E1 osteogenesis. MC3T3-E1 cells were firstly transfected with nc-siRNA or verified Atf7ip-siRNA and cultured in 500 ng/mL PTH for the indicated time. (**A**) Western blot with specific antibodies for Setdb1, H3K9me3, and H3. Gapdh was used as the loading control, *n* = 3. (**B**–**D**) mRNA levels of *Alp*, *Col1a1*, and *Osteocalcin* examined by real-time PCR, *n* = 3. The results of alkaline phosphatase staining (**E**), the Alp activity assay (**F**), and Alizarin red staining (**G**). Scale bar, 100 μm. The experiments were repeated at least three times. Data are expressed as mean ± SD. The significance was measured by one-way ANOVA, * *p* < 0.05, ** *p* < 0.01, *** *p* < 0.001, **** *p* < 0.0001, treatments vs. no treatment; ^#^ *p* < 0.05, ^##^ *p* < 0.01, ^###^ *p* < 0.001, ^####^ *p* < 0.0001, Atf7ip-siRNA vs. nc-siRNA.

To verify the role of Atf7ip in osteoblast differentiation, Atf7ip was knocked down with specific siRNA, and the osteoblast differentiation was then analyzed. Atf7ip–siRNA transfection significantly inhibited the mRNA and protein levels of Atf7ip (Figure S3A,B). The cell viability was comparable between the Atf7ip interference cells and the controls (Figure S3C). At the protein level, Atf7ip knockdown decreased the level of H3K9me3 in the PTH-treated cells, but the Setdb1 expression was unchanged (Figure 2A). Unexpectedly, the *Alp*, *Col1a1*, and *Osteocalcin* expressions induced by PTH were further elevated in the Atf7ip–siRNA-treated cells (Figure 2B–D). Furthermore, both the Alp-positive cells and Alp activity increased in the Atf7ip–siRNA groups relative to the nc-siRNA-treated cells (Figure 2E,F). In accordance with the staining results, the calcium deposition level significantly increased in the Atf7ip–siRNA cells (Figure 2G).

### 2.3. Atf7ip Overexpression Inhibits the Osteogenic Differentiation of MC3T3-E1 Cells

**Figure 3:**
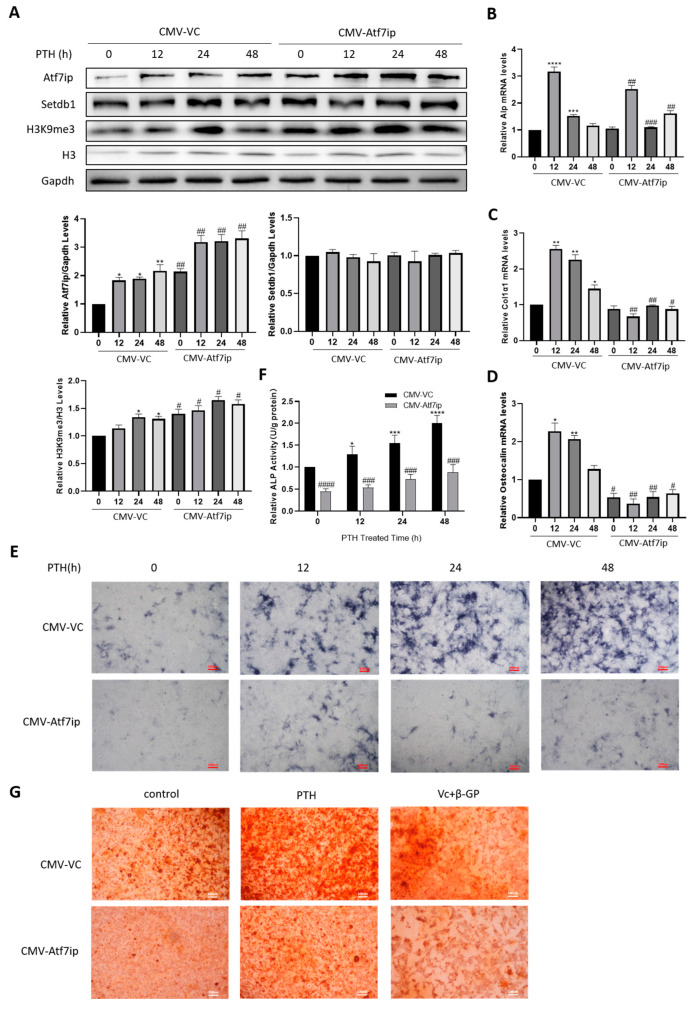
Overexpression of Atf7ip inhibits the osteogenic differentiation of MC3T3-E1 cells. The cells were first transiently transfected with the Atf7ip overexpression vector (CMV-Atf7ip) or its vector control (CMV-VC) for 24 h, and were then treated with 500 ng/mL PTH for the indicated time. The protein (**A**) and RNA (**B**–**D**) were collected to analyze the expression levels of the target molecules. *n* = 3. Alkaline phosphatase staining (**E**) and Alp activity assay (**F**). (**G**) Alizarin red staining of Atf7ip overexpression cells with PTH or osteoblast differentiation culture for 7 days. The experiments were repeated at least three times. Scale bar, 100 μm. Data are expressed as mean ± SD. The significance was measured by one-way ANOVA, * *p* < 0.05, ** *p* < 0.01, *** *p* < 0.001 **** *p* < 0.0001, treatments vs. no treatment; ^#^*p* < 0.05, ^##^ *p* < 0.01, ^###^ *p* < 0.001, ^####^ *p* < 0.0001, CMV-Atf7ip vs. CMV-VC.

We then transfected MC3T3-E1 cells with an Atf7ip overexpression vector (CMV-Atf7ip). At the protein level, the CMV-Atf7ip cells expressed more Atf7ip (Figure 3A). The H3K9me3 level consistently significantly increased in the CMV-Atf7ip cells, although the Setdb1 protein level was not altered (Figure 3A). Firstly, Atf7ip overexpression did not affect the cell viability, as indicated by a CCK-8 assay. We further measured the osteoblast differentiation markers in CMV-Atf7ip cells with or without PTH treatment. In the CMV-Atf7ip cells, the mRNA levels of *Alp*, *Osteocalcin*, and *Col1a1* induced by PTH were inhibited in comparison with the CMV-VC cells (Figure 3B–D). An Alp staining and Alp activity assay demonstrated that Atf7ip overexpression inhibited the osteoblast differentiation of the PTH-treated MC3T3-E1 cells (Figure 3E,F). Calcium deposition was also suppressed in the CMV-Atf7ip cells, as reflected by Alizarin red staining (Figure 3G). Therefore, these data suggest that Atf7ip overexpression effectively attenuates the osteoblast differentiation of MC3T3-E1 cells.

### 2.4. Osteoblastic Atf7ip Deficiency Mice Show Higher Bone Formation

**Figure 4:**
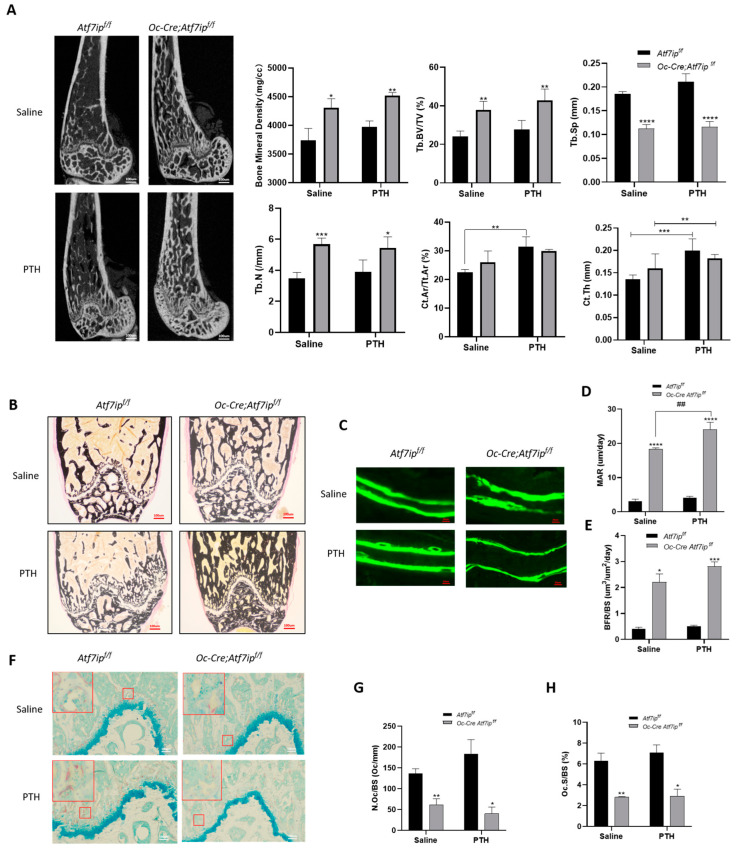
Osteoblastic Atf7ip deficiency mice show higher osteoblastic bone formation. (**A**) Representative micro-CT images and calculated parameters of the femoral metaphysis of male *Oc-Cre;Atf7ip^f/f^* (2 months old, *n* = 6) and littermate control mice (*Atf7ip^f/f^*, *n* = 4) with or without PTH treatment (80 μg/kg b w, 5 days per week for 4 weeks). μ-CT scan, located at 0.5 cm on the distal femur. BMD, bone mineral density; Tb.BV/TV, trabecular bone volume/total volume; Tb.Sp, trabecular space; Tb.N, trabecular number; Ct.Ar/Tt.Ar, cortical area/total area; Ct.Th, cortical thickness. (**B**–**H**). Von Kossa staining, Scale bar, 100 μm. (**B**), double calcein staining, Scale bar, 20 μm. (**C**), and TRAP staining, Scale bar, 100 μm. Selected sections magnified 10-fold (**F**) of undecalcified sections of the femurs of *Oc-Cre;Atf7ip^f/f^* and *Atf7ip^f/f^* mice. Representative images are shown. (**D**,**E**) Dynamic histomorphometry analysis of the trabecular bone from the femoral metaphysis of *Oc-Cre;Atf7ip^f/f^* and *Atf7ip^f/f^* mice based on the double calcein staining images. MAR, mineral apposition rate. BFR/BS, bone formation rate/bone surface. (**G**,**H**) Statics of osteoclast parameters based on the TRAP staining images. N.Oc/Bs, number of osteoclasts/bone surface; Oc.s/Bs, osteoclast surface/bone surface. Data are expressed as mean ± SD. *n* = 5. The significance was measured by one-way ANOVA, * *p* < 0.05, ** *p* < 0.01, *** *p* < 0.001, **** *p* < 0.0001, ^##^ *p* < 0.01. *Oc-Cre;Atf7ip^f/f^* vs. *Atf7ip^f/f^*.

To investigate the role of osteoblastic Atf7ip in bone formation in vivo, we generated osteoblastic-*Atf7ip*-specific knockout (*Oc-Cre;Atf7ip^f/f^*) mice by crossing *Atf7ip^Flox/Flox^* mice (*Atf7ip^f/f^*) with *Osteocalcin-Cre* mice (*Oc-Cre*) (Figure S4A). A PCR test of the DNA templates from tissues of the *Oc-Cre;Atf7ip^f/f^* mice verified that Cre-modulated recombination occurred exclusively in the bone (Figure S4B). The body length and weight were comparable between the *Oc-Cre;Atf7ip^f/f^* mice and their control littermates. Moreover, there was no difference in the length of the femurs (Figure S4C). Micro-CT of the proximal femurs showed the trabecular bone, trabecular bone volume/total volume, trabecular number, and trabecular space were improved in the *Oc-Cre;Atf7ip^f/f^* mice compared to their littermates (Figure 4A). In the cortical bone, the cortical area and cortical thickness were comparable between the *Oc-Cre;Atf7ip^f/f^* mice and the controls (Figure 4A). Moreover, Von Kossa staining showed greater mineral deposition in the *Oc-Cre;Atf7ip^f/f^* mice (Figure 4B). The new bone formation was further validated by double calcein labeling. An analysis of the histological sections demonstrated that *Atf7ip* osteoblastic deficiency increased the bone mineral deposition rate as well as the bone formation rate (Figure 4C–E). Regarding the spine, the *Oc-Cre;Atf7ip^f/f^* mice had a higher bone density and bone volume compared to the control mice (Figure S5A). RNA-seq was further used to measure changes at the molecular level. The results showed that genes involved in extracellular matrix organization, collagen formation, and collagen biosynthesis were enriched in the bones of the *Oc-Cre;Atf7ip^f/f^* mice (Figure S5B).

The effect of *Atf7ip* on PTH-induced bone formation was also evaluated by micro-CT and histomorphometry (Figure 4A). In the control mice, the PTH treatment significantly improved the cortical bone parameters, but did not lead to changes in the volume, number, or thickness of the trabecular bone (Figure 4A). In the *Oc-Cre;Atf7ip^f/f^* mice, these trabecular parameters were further improved upon PTH treatment, but *Atf7ip* deficiency in the osteoblasts had no effect on the cortical parameters mediated by PTH treatment (Figure 4A). Von Kossa staining and double calcein labeling showed that the PTH-treated *Oc-Cre;Atf7ip^f/f^* mice showed an increase in bone formation (Figure 4B–E). Interestingly, the PTH-treated *Oc-Cre;Atf7ip^f/f^* mice showed less osteoclast formation, although the PTH treatment increased the osteoclast number in their control littermates (Figure 4F–H), indicating that bone coupling was inhibited in the *Oc-Cre;Atf7ip^f/f^* mice. Taken together, the results indicate that *Atf7ip* osteoblastic deficiency enhances bone formation and mineral apposition.

### 2.5. Expression of Atf7ip Influences the Localization of Setdb1 in the Nucleus

**Figure 5:**
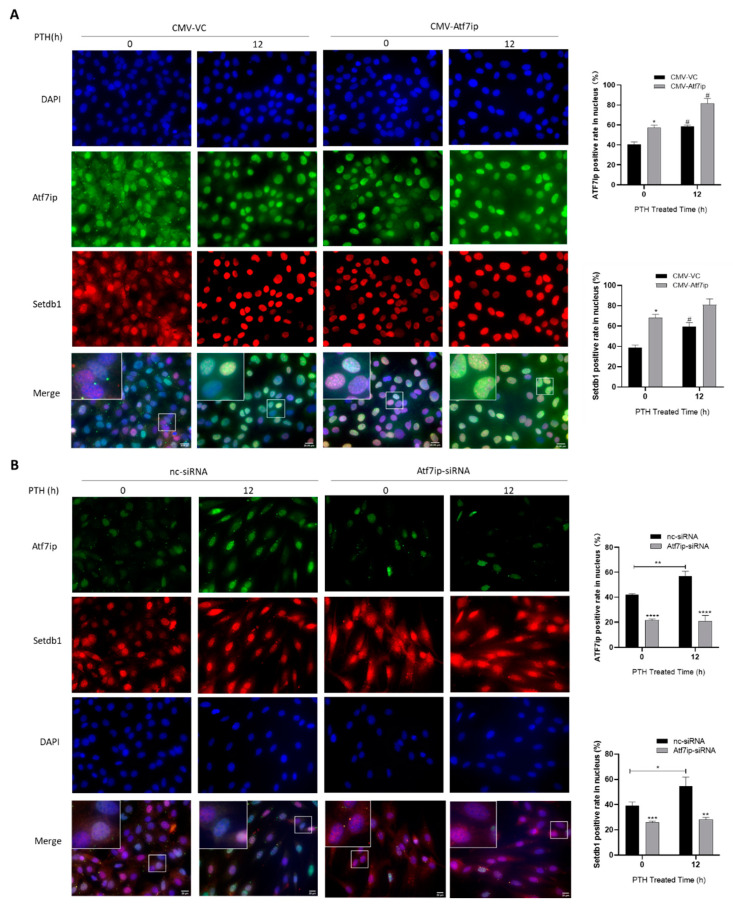
Expression of Atf7ip influences the nucleus localization of Setdb1 in MC3T3-E1. MC3T3-E1 cells were first transfected with CMV-Atf7ip (**A**) or Atf7ip-siRNA (**B**) and their respective controls, and were then treated with or without 500 ng/mL PTH for 12 h. The localization of Atf7ip (green) and Setdb1 (red) was examined by immunofluorescence staining. The nucleus was verified by DAPI (blue). Representative images and high magnification for representative images are shown. Scale bar, 20 μm. Selected sections magnified 10-fold. The cells with nucleus localization of Atf7ip or Setdb1 were calculated by Image J, *n* = 4. The experiments were repeated at least three times. Data are expressed as mean ± SD. The significance was measured by one-way ANOVA, * *p* < 0.05, ** *p* < 0.01, *** *p* < 0.001, **** *p* < 0.0001, Atf7ip overexpression or interference vs. vector control or nc-siRNA group; ^#^ *p* < 0.05, PTH group vs. saline group.

Atf7ip functions as a binding partner of Setdb1, which was previously found to be involved in osteoblast differentiation. Recently, Zhang et al. found that Atf7ip regulated Setdb1 nuclear localization and affected osteoblast proliferation [20]. To investigate the mechanism of this regulation of Atf7ip in regard to osteoblast differentiation, we first measured whether Atf7ip affected Setdb1 nuclear localization in the osteoblasts. The immunofluorescence assays showed that the nuclear localization of Setdb1 increased when Atf7ip was forcibly expressed in MC3T3-E1 cells (Figure 5A). Upon the silencing of Atf7ip with siRNA, Setdb1 expression in the nucleus decreased (Figure 5B). These results are consistent with the findings of a recent report [20]. Moreover, the PTH treatment increased Setdb1 nuclear localization, which was further enhanced in the Atf7ip-overexpressing cells (Figure 5A). The Setdb1 in the nucleus, mediated by PTH treatment, was also inhibited in the Atf7ip-siRNA cells (Figure 5B). The results suggest that Atf7ip affects the nuclear localization of Setdb1, which, in turn, affects its regulation of osteoblast differentiation.

### 2.6. Inhibition of Sp7 Inhibits Atf7ip–siRNA-Promoting Bone Differentiation

**Figure 6:**
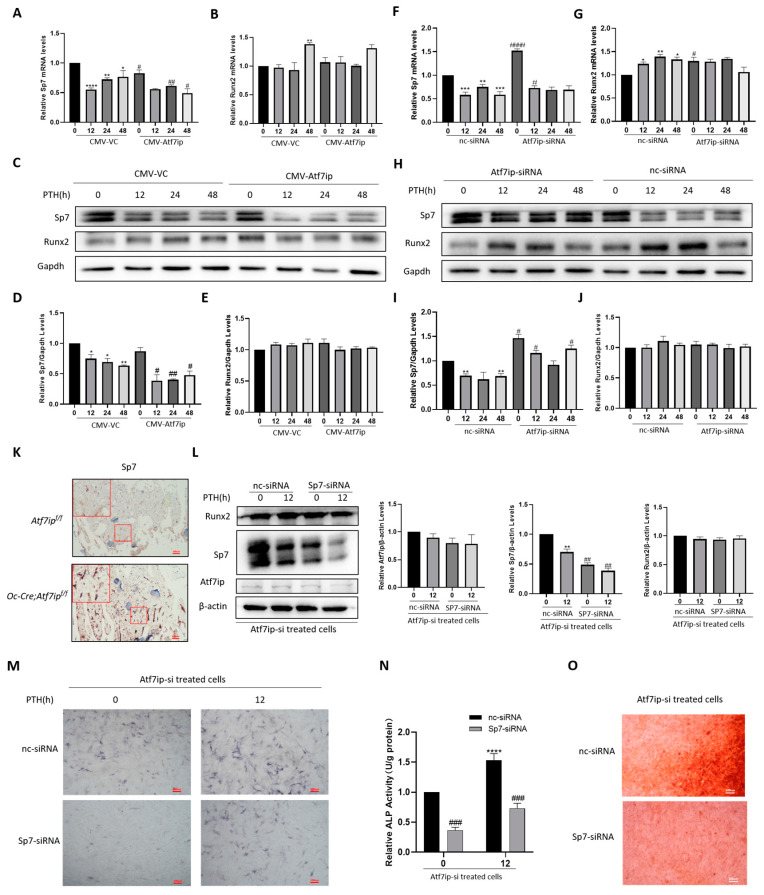
Inhibition of Sp7 attenuates Atf7ip–siRNA-promoting osteoblast differentiation. MC3T3-E1 cells were first transfected with CMV-Atf7ip (**A**–**E**) or Atf7ip-siRNA (**F**–**J**) and their respective controls, and then treated with 500 ng/mL PTH for the indicated time. The mRNA and protein levels of Runx2 and Sp7 were analyzed by q-RT-PCR and Western blot, respectively. (**K**) Immunohistochemistry of Sp7 in the femoral section of Ocn-Cre Atf7ip^fl/fl^ and Atf7ip^fl/fl^ mice. The high magnification for representative images is shown. Selected sections magnified 10-fold. Scale bar = 100 μm (**L**–**O**) MC3T3-E1 cells were treated with Atf7ip-siRNA for 24 h, followed by transient nc-siRNA and Sp7-siRNA for 24 h and, finally, PTH for 12 h. (**L**) Western blot of the protein expressions of Runx2, Sp7, and Atf7ip. ALP staining (**M**), ALP activity assays (**N**), and ARS staining (**O**) were used to detect the osteoblast differentiation status. For WB, Gapdh was used as the loading control. The experiments were repeated at least three times. For ALP staining and ARS staining, scale bar = 100 μm. Data are expressed as mean ± SD (*n* = 3). The significance was measured by one-way ANOVA, * *p* < 0.05, ** *p* < 0.01, *** *p* < 0.001, **** *p* < 0.0001, PTH group vs. saline group; ^#^ *p* < 0.05, ^##^ *p* < 0.01, ^###^ *p* < 0.001, ^####^ *p* < 0.0001. overexpression or interference vs. vector control or nc-siRNA group.

Setdb1 affected osteoblast differentiation via its regulation of the activity and expression of transcription factors, such as Runx2 and Sp7 [2,9]. We first measured whether Atf7ip affected the expression of Runx2 and Sp7. In the Atf7ip-overexpressing cells, Sp7 expression was restricted, but the Runx2 protein level was unchanged (Figure 6A–E). In contrast, Sp7 increased in the Atf7ip–siRNA cells, and the level of Runx2 was comparable to the control (Figure 6F–J). In the tibia sections of the *Oc-Cre;Atf7ip^f/f^* mice, the immunostaining intensity of Sp7 also increased (Figure 6K). PTH induced Runx2 expression, which cannot be affected by Atf7ip (Figure 6A–J). However, the inhibition of Sp7 upon PTH treatment was partially mitigated in the Atf7ip knockdown cells and was further suppressed when Atf7ip was overexpressed (Figure 6A–J). To confirm whether Sp7 played a key role in osteoblast differentiation mediated by Atf7ip, Sp7 was knocked down in MC3T3-E1 cells pretreated with Atf7ip–siRNA (Figure 6L). Alp staining and Alizarin red staining showed that both the osteogenesis capacity and mineralization density decreased in the Sp7-siRNA cells compared to the control group (Figure 6M–O).

## 3. Discussion

The relationship between histone epigenetic modification and bone homeostasis maintenance has been extensively studied in the last decade [5,20]. Some key HPTMs and their regulatory proteins have been found to play critical roles in osteoblast lineage commitment [5,20]. Here, we proved that Atf7ip, a conserved cofactor of Setdb1, functions as a negative regulator of osteoblast differentiation, with the findings from an Atf7ip-dysregulated cellular model in vitro and mice with osteoblastic-Atf7ip-specific deficiency in vivo. Firstly, the evidence showed that Atf7ip was upregulated in differentiated osteoblasts. This expression pattern appeared to be non-cell-autonomous, as it was observed in different osteoblast lineages and was identified following the use of various types of stimuli, such as PTH and an osteogenic medium with β-glycerophosphate and ascorbic acid. We then demonstrated that Atf7ip was a negative regulator of osteoblast differentiation in the context of Atf7ip overexpression or knockdown in pre-osteoblast MC3T3-E1. This negative role in the regulation of osteoblast differentiation was further confirmed by the bone density analysis and bone histomorphometry experiments on *Oc-Cre;Atf7ip^f/f^* mice.

Atf7ip functions as a regulator of H3K9me3 modification to control the expression and activity of Setdb1 [10,11,14,15]. There is evidence demonstrating that H3K9 methylation and Setdb1 are involved in the regulation of osteoblast differentiation [5]. Moreover, Setdb1 promotes osteoblast proliferation by repressing ADP-ribose glycohydrolase Macrod2 [17]. In the current study, we found that the H3k9me3 level was positively regulated by Atf7ip. Nevertheless, Setdb1 expression was not altered in the Atf7ip-dysregulated cells. We then measured the effect of Atf7ip on the cellular localization of Setdb1, as the nucleus localization of Setdb1 was important for its function. We found that Atf7ip positively regulated Setdb1 localization in the nucleus, which is consistent with the findings of Zhang et al. [17]. Atf7ip might regulate osteoblast differentiation by mediating Setdb1 nucleus localization, thereby increasing the activity and methylation level of H3K9. A further study, in which the authors designed small molecular or antagonist peptides, showed that interrupting the Atf7ip–Setdb1 complex could contribute to efforts to answer this question [21].

**Figure 7:**
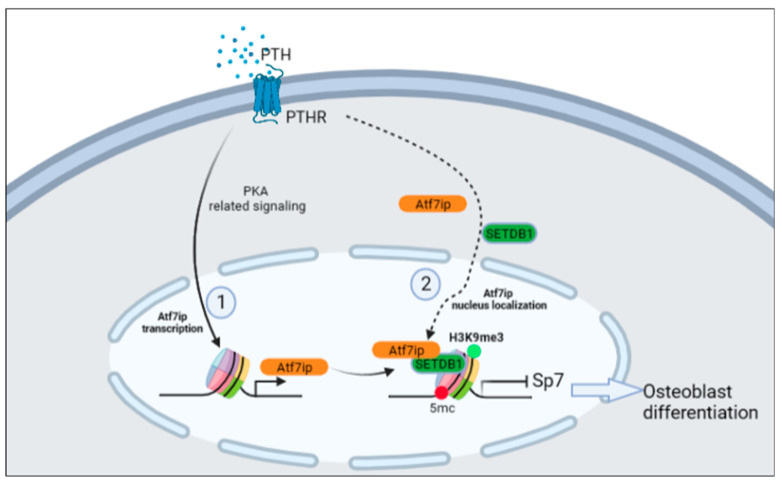
The figure abstractly reviews the mechanisms underlying the negative regulatory effect of Atf7ip on osteoblast differentiation. Atf7ip is upregulated by PTH–PKA-related signaling in MC3T3-E1 cells and regulated the nuclear localization of Setdb1, which further inhibited Runx2 activity and Sp7 expression, thereby inhibiting osteoblast differentiation.

Setdb1 inhibited osteoblast differentiation by regulating Runx2 activity, but not its expression [9]. In the current study, Runx2 expression was also unchanged in both the Atf7ip-overexpressing and Atf7ip-siRNA-treated cells. Additionally, Setdb1 regulated the expression of Sp7, a key transcription factor (TF) for osteoblast differentiation [2]. We found that Sp7 expression was negatively regulated by Atf7ip in pre-osteoblast models. Furthermore, Sp7 knockdown partially reversed the osteoblast differentiation phenotype in the Atf7ip-deficient cells. Thus, with the current evidence and the literature, we can suggest that Atf7ip might regulate osteoblast differentiation by controlling Setdb1 localization, thereafter affecting Runx2 activity and Sp7 expression (Figure 7). Atf7ip knockdown led to impaired osteoblast proliferation [17], since cell-cycle exit contributed to the termination of osteoblast differentiation [22,23,24]. It is probable that the osteoblast differentiation induced by the interference of Atf7ip was partly due to its limited proliferation status. However, in our model, Atf7ip expression did not affect the cell viability in either the Atf7ip-overexpressing or Atf7ip knockdown cells, which is inconsistent with a recent finding [17]. A possible reason for this discrepancy is that the cell density used in the current study was much higher than the reported one.

H3K9 methylation helps to maintain cell identity by preventing the inappropriate expression of tissue-specific genes [6]. In mammal tissue, the status of H3K9 methylation is mediated by the KMTs Suv39H1/2, Setdb1, and G9a, as well as KDMs such as LSD1/Kdm1 and KDM4a-d. The role of H3K9 methylation in the osteoblast differentiation of multipotency mesenchymal stem cells was verified by targeting the KMT or KDM on pluripotency stem cells within the Cre-LoxP system in vivo (e.g., Prrx1 Cre, Sox-9 Cre) [9,25,26], or by interfering with KMT or KDM expression in vitro on either primary bone marrow mesenchymal stem cells or the established cell lines known as pluripotency C3H/10T1/2 cells and ST2 [27,28]. The deficiency of G9a or Setdb1 in pluripotency stem cells led to hypo-H3K9 methylation and impaired osteoblast differentiation, which might be related to the irregulated Runx2 activity [9,25]. The mesenchymal-targeted knockdown of LSD1, a KDM for H3K4, H3K9, and H3K27, resulted in lower osteoblast activity and damaged primary spongiosa ossification and reorganization in vivo [26]. However, LSD1 inhibition reportedly enhanced the osteogenic division of MSCs [29,30]. KDM4A demethylates lysine 9 (H3K9me2/3) and lysine 36 (H3K36me3), although the demethylation of lysine 9 (K9) occurs preferentially and is more efficient than the demethylation of lysine 36 (K36) [31,32]. KDM4a overexpression in ST2 cells enhanced adipogenesis, but impaired osteoblastic genesis [28]. However, Qin et al. found that transiently silenced KDM4A inhibited BMSC osteoblast differentiation [27]. Thus, the role of H3K9 methylation in the osteoblast differentiation of multipotency stem cells remains controversial. Epigenetic alteration affects bone formation in a stage-dependent manner [33]. Based on our observations using the pre-osteoblast MC3T3-E1 cell model and mature osteoblast Atf7ip-deficient mouse model, we found that Atf7ip, the H3K9me3 modulator, controlled osteoblastogenesis in the later developmental stages, which indicated the negative role of H3K9 methylation in osteoblast differentiation. However, as *Oc-Cre* also targets broader stomal cell populations [34], the role of Atf7ip in mature osteoblasts should be interpreted in more detail and further verified in other models, such as *Col1A1-Cre* [35].

Our study further ascertained the role of Atf7ip in the effect of PTH on bone formation. PTH is an important regulator of bone homeostasis that functions by modulating osteoblast proliferation, differentiation, and apoptosis resistance through PTHR signaling [18]. However, sustained PTH can lead to catabolic effects, such as the inhibition of Sp7 expression [19] and promotion of RANKL expression [36]. In the current study, PTH enhanced Atf7ip expression in a PKA-dependent manner. Atf7ip knockdown decreased the H3K9me3 levels in the PTH-treated cells and diminished the suppressing effect of PTH on Sp7 expression. Our findings suggest that Atf7ip is a potential regulator of the catabolic effect of PTH. These data support the notion that histone epigenetic modification might be an important regulatory mechanism of the catabolic effect of PTH.

In summary, in this study, Atf7ip functioned as a suppressor of osteoblast maturation and negatively regulated osteoblast differentiation by regulating Setdb1 cellular localization and inhibiting Sp7 expression in the osteoblasts. This mechanistic characterization provides us with insight into the ways in which H3K9 modification regulates osteoblast differentiation in committed osteoblasts.

## 4. Materials and Methods

### 4.1. Reagents and Antibodies

Alpha-modified essential medium (α-MEM) and trypsin/EDTA were purchased from Gibco (Thermo Fisher Scientific, Waltham, MA, USA). Penicillin–streptomycin solution was obtained from Beyotime Biotechnology (Shanghai, China). Dimethyl Sulfoxide (DMSO), insulin, dexamethasone, isobutyl methylxanthine (IBMX), and indomethacin were purchased from Sigma-Aldrich (Sigma-Aldrich, St. Louis, MO, USA). Parathyroid hormone (hPTH_1–34_, H-4835) was from BACHEM Inc. (Torrance, CA, USA). Lipofectamine^TM^ 2000 was purchased from Invitrogen™ (Thermo Fisher Scientific). The antibody for Atf7ip was obtained from Santa Cruz Biotechnology Inc. (Santa Cruz Biotechnology, Inc., Santa Cruz, CA, USA). The antibodies for H3, Runx2, Gapdh and Setdb1 came from Proteintech Group (Rosemont, IL, USA). The antibodies for H3K9me3, Sp7 and β-actin were acquired from Abcam (Cambridge, MA, USA).

### 4.2. Cell Culture

MC3T3-E1 cells (MC3T3-E1 Subclone 14) and C3H/10T1/2 (Clone 8) were purchased from the National Collection of Authenticated Cell Cultures (Shanghai, China) and maintained in α-MEM supplemented with 100 U/mL penicillin–streptomycin (Beyotime) and 10% fetal bovine serum (FBS, Every Green, ZheJiang TianHang Biotechnology Co., Ltd., Hangzhou, China). Bone marrow stromal cells (BMSCs) were harvested and cultured as reported [37]. Briefly, long bones were first separated from C57BL/6J mice aged 2–3 months. Then, small cuts were made at both the proximal and distal ends of the bones, and a syringe was used to flush the cells. The cells were collected, centrifuged, and suspended in α-MEM with 10% FBS and 1% penicillin–streptomycin, then plated in cell culture dishes (1.0 × 10^6^ cells/cm^2^) for 72 h. The cell culture medium that contained the suspended cells was removed, and the adherent BMSCs were used for further analysis.

### 4.3. Generation of Atf7ip Conditional Mouse Strains and In Vivo Parathyroid Hormone Treatment

Floxed *Atf7ip* mice (*Atf7ip^Flox/Flox^*) were created from an embryonic stem (ES) cell clone HEPD0922-3 from the International Mouse Phenotyping Consortium (IMPC) as described [38]. The strain was cryorecovered by the CAM-CU Genomic Resource Center and kindly gifted by Dr. Jian Chen (Chinese Institute for Brain Research, Beijing, China). To obtain osteoblasts with specific *Atf7ip* knockout, we purchased *Osteocalcin-Cre* mice (Ocn-Cre, Cyagen Biosciences, strain#C001025) and crossbred them with *Atf7ip^Flox/Flox^* (Figure S4A). Genomic PCR primers were used to identify *Ocn-Cre;Atf7ip^Flox/Flox^* (referred to as *Oc-Cre;Atf7ip^f/f^*) mice (forward and reverse primer pairs: *Atf7ip loxP*: 5′-AGGCTTGTTGGCAAGTGTCT-3′, 5′-CACTGAGTCTCTGGCATCTC-3′; *Ocn-Cre*: 5′-CAAATAGCCCTGGCAGATTC-3′, 5′-TGATACAAGGGACATCTTCC-3′). The tissue-specific deletion of *Atf7ip* was also confirmed at the RNA level by real-time quantitative polymerase chain reaction (RT-qPCR) using RNA isolated from the heart, liver, lung, kidney, and femur of the *Oc-Cre;Atf7ip^f/f^* mice (Figure S4B). For parathyroid hormone treatment, male mice aged 6–8 weeks were intermittently administered with a subcutaneous injection of either saline vehicle or hPTH_1–34_ (80 μg/kg b w, 5 days a week for 4 weeks). All procedures involving animals were conducted in the Animal Facility of Jiangnan University School of Medicine. The animal protocols were reviewed and approved by the Animal Care and Ethics Committee of Jiangnan University (Wuxi, Jiangsu, China).

### 4.4. siRNA-Mediated Knockdown

MC3T3-E1 cells (3 × 10^4^ cells/cm^2^) were placed in α-MEM with 10% FBS and 100 U/mL penicillin–streptomycin. On the next day, the cells were transfected with siRNAs using Lipofectamine 2000 transfection reagent (Thermo Fisher Scientific) in standard α-MEM. Atf7ip-siRNA was bought from Dharmacon. Dharmacon smart-pool siRNAs (GE Healthcare) targeting Atf7ip (Cat: M-042275-00-0005) and the nontargeting control (Cat: D-001206-13-05) were used for a final content of 10 nM. Sp7-siRNA (Lot: N0612, 5′-TCCAAGCGCTTTACCAGAA-3′) and its negative control (Lot: S1012; nc-siRNA: 5′-GGCTCTAGAAAAGCCTATGC-3′) were purchased from Ruibo (Guangzhou RiboBio Co., Ltd., China).

### 4.5. Construction of Atf7ip Plasmid

The expression constructs of Atf7ip (CMV-Atf7ip, 200 ng/μL) (Cat: GOSE0316729) and empty vector (CMV-VC, 200 ng/μL) (Cat: P21122600) were purchased from Gene Biotechnology (GENECHEM, Shanghai, China). The Atf7ip expression plasmid and empty vector were transiently transferred with Lipofectamine 2000 transfection reagent (Thermo Fisher Scientific) to induce Atf7ip overexpression in MC3T3-E1 cells.

### 4.6. Osteogenic Differentiation

For experiments requiring osteogenic differentiation, MC3T3-E1 cells (3.0 × 10^4^ cells/cm^2^) were planted on standard 12-well tissue culture plates with standard α-MEM (10% FBS, 100 U/mL penicillin-streptomycin). On the next day, the maintenance media were substituted with osteogenic media consisting of a standard medium with 500 ng/mL PTH or osteogenic cocktail added (50 µg/mL ascorbic acid (Vc), 10 mM β-glycerol phosphate (β-Gp)) for the indicated days. Vehicle groups were supplemented with equivalent concentration of DMSO, if appropriate. The medium was refreshed every 3 days and replenished with PTH or osteogenic cocktail.

### 4.7. Adipogenic Differentiation

To initiate adipogenic differentiation, BMSCs or C3H/10T1/2 cells were processed with an adipogenic medium containing 5 μg/mL insulin, 0.5 μM dexamethasone, 0.25 mM IBMX, and 50 μM indomethacin. After 7 days of culture, the adipogenic cells were detected using an oil red staining kit (Solarbio, Beijing, China).

### 4.8. RNA Extraction and q-PCR

TRIzol reagent (TaKaRa, Tokyo, Japan) was used to extract total RNA from MC3T3-E1 cells, C3H/10T1/2 cells, or BMSCs, and was reverse-transcribed to cDNA by PrimeScript^TM^ RT Master Mix (Thermo Fisher Scientific). Then, qPCR was performed by a Lightcycler 480II system (Roche, Manneim, Germany) with ChamQ SYBR Color qPCR Master Mix (Vazyme Biotech Co., Ltd., Nanjing, China), following the instructions provided, to test the expressions of *Atf7ip, Setdb1, Alp, Runx2, Sp7, Osteocalcin, Col1a1, Pparγ, Twist, Cebp/α,* and *β-actin*. The primers for these genes were retrieved from PrimerBank [39], and are listed in Table 1. Each sample was analyzed in triplicate and data were standardized to the β-actin level (ΔΔC_T_).

### 4.9. Western Blot

BMSCs and MC3T3-E1 cells were treated in a RIPA lysis buffer (Beyotime) with protease and phosphatase inhibitors (Cell Signaling Technology, Inc., Danvers, MA, USA). Then, the cells were subjected to 6–12% sodium dodecyl sulfate-polyacrylamide gel electrophoresis (Sinopharm, Shanghai, China) and removed to polyvinylidene fluoride membranes (Merck KGaA, Darmstadt, Germany). The membranes were blocked with 5% non-fat milk in a TBST buffer and incubated at 4 ℃ overnight with specific primary antibodies. The Atf7ip antibody was diluted to 1:100. The antibodies for Setdb1, Sp7, Runx2, H3, and H3K9me3 were diluted to 1:2000. The enzyme-conjugated secondary antibody (Jackson ImmunoResearch Laboratories, PA, USA) was added and detected by an enhanced chemiluminescence (ECL) system (Tanon Technology Co., Ltd., Shanghai, China). Densitometry was conducted on ImageJ (Bethesda, MD, USA).

### 4.10. Immunofluorescence Staining

MC3T3-E1 cells were fixed with 4% paraformaldehyde at room temperature for 15 min, and then blocked in a QuickBlock™ blocking buffer (Beyotime) for 1 h. Then, the cells were cultured, first with specific primary antibodies overnight and then with FITC- or Alexa-conjugated secondary antibodies for 2 h. To detect nuclei, the cells were stained with antifade mounting medium with DAPI (Vector Laboratories Inc., Newark, CA, USA) and examined by an Axio imagerM2 fluorescence microscope (Carl Zeiss AG, Germany).

### 4.11. Alkaline Phosphatase (Alp) Staining and ALP Activity Assay

MC3T3-E1 cells (3 × 10^4^ cells/cm^2^) were cultured on 24-well plates and treated by ALP staining with TRACP and an ALP double-staining kit (TaKaRa). For the ALP activity assay, MC3T3-E1 cells (1 × 10^4^ cells/well) were cultured on 96-well plates and analyzed with an alkaline phosphatase assay kit (TaKaRa).

### 4.12. Alizarin Red Staining

MC3T3-E1 cells (3 × 10^4^ cells/cm^2^) were cultured on 12-well plates, cleaned with PBS twice, and fixed with 4% paraformaldehyde (Sigma) for 15 min. After washing with PBS twice, the cells were incubated with an Alizarin red solution (Merck Millipore) for 30–60 min. After that, the cells were washed with PBS twice and imaged with a CKX53 Microscope (Olympus Corporation, Tokyo, Japan).

### 4.13. Immunohistochemical Staining

The dissected femurs were fixed in 4% polyoxymethylene for 3 days and decalcified in 7.5% EDTA for 6 months before sectioning (5 μm). Slides were subjected to sodium citrate buffer at 99 °C for 1 h for antigen retrieval and then grown with mouse anti-Sp7 (1:50, sc-100631, Santa Cruz).

### 4.14. Calcein Double Labeling and Von Kossa Staining

Mice were peritoneally injected with 50 mg/kg calcein 7 and 3 days prior to execution. The femurs were then immobilized in 4% PFA for 48 h and encased in methacrylate without decalcification. The samples were sliced with sliding slicers, observed, and photographed with fluorescent microscopy. Dynamic bone formation was measured by mineral apposition rate (MAR) on the bone surface and bone formation rate (BFR/BS) per unit of bone surface, as previously indicated. Calculations were made in five randomly selected regions from the epiphysis of the distal femur. For the von Kossa staining assay, the sample was sliced with a sliding slicer and then dyed as per the calcium stain kit (Von Kossa Method) (Solarbio, Cat: G3282).

### 4.15. Micro-CT

After fixation in 4% paraformaldehyde, the femurs and spines from both the *Oc-Cre;Atf7ip^f/f^* mice and the control mice were scanned by a Quantum GX micro-CT system (PerkinElmer, MA, USA) at a voltage of 90 kV, a current of 88 μA, and a resolution of 12 μm per pixel. A bone region of interest (ROI) was drawn from 0.15 mm proximal to the distal epiphyseal growth plate and extended proximally for 5.4 mm to determine bone volume fraction (BV/TV), trabecular thickness (Tb.Th), trabecular number (Tb.N), trabecular separation (Tb.Sp), cortical area fraction (Ct.Ar/Tt.Ar), and mean cortical thickness (Ct.Th).

### 4.16. Cell Proliferation

Cells were planted at 1.0× 10^4^ cells/well in 96-well plates and transfected with Atf7ip expression plasmid or vector using the jetPRIME transfection reagent (PolyPlus, Berkeley, CA, USA). After 24 h, the cell growth rate was determined using a cell counting kit-8 (APExBIO, Houston, TX, USA).

### 4.17. Statistical Analysis

Data are represented as mean ± standard deviation (SD). Significance at *p* < 0.05 was recognized via two-tailed Student’s *t*-test or one-way analysis of variation (ANOVA), followed by Newman–Keuls post hoc tests if appropriate.

## Figures and Tables

**Table 1:** Sequences of PCR primers used in RT-qPCR.

Gene	Primer Sequences (5′→3′)
Mouse *Atf7ip*	FP: CGGGAGCCATGAGAATGGAG
	RP: GCATACAAGGGGTCTCTTTCC
Mouse *Alp*	FP: CCAACTCTTTTGTGCCAGAGA
	RP: GGCTACATTGGTGTTGAGCTTTT
Mouse *Runx2*	FP: ATGCTTCATTCGCCTCACAAA
	RP: GCACTCACTGACTCGGTTGG
Mouse *Sp7*	FP: CCTCTGCGGGACTCAACAAC
	RP: AGCCCATTAGTGCTTGTAAAGG
Mouse *Osteocalcin*	FP: CTGACCTCACAGATCCCAAGC
	RP: TGGTCTGATAGCTCGTCACAAG
Mouse *Setdb1*	FP: CCTGGGTGCATGAGTTTGG
	RP: TGTACTGACGAAGTTCCTCCATA
Mouse *Col1a1*	FP: GCTCCTCTTAGGGGCCACT
	RP: CCACGTCTCACCATTGGGG
Mouse *β-actin*	FP: GGCTGTATTCCCCTCCATCG
	RP: CCAGTTGGTAACAATGCCATGT
Mouse *Pparγ*	FP: TCGCTGATGCACTGCCTATG
	RP: GAGAGGTCCACAGAGCTGATT
Mouse *Cebp/α*	FP: CAAGAACAGCAACGAGTACCG
	RP: GTCACTGGTCAACTCCAGCAC
Mouse *Twist*	FP: GGACAAGCTGAGCAAGATTCA
	RP: CGGAGAAGGCGTAGCTGAG

## Data Availability

Data will be made available on request wuyu@jiangnan.edu.cn.

## Supplementary Materials

The following supporting information can be downloaded at: https://www.mdpi.com/article/10.3390/ijms24054305/s1.
