# Supplementary material for: Atf7ip Inhibits Osteoblast Differentiation via Negative Regulation of the Sp7 Transcription Factor
Source: Int J Mol Sci. 2023 Feb 21;24(5):4305. doi: 10.3390/ijms24054305 (PMC10002255; doi:10.3390/ijms24054305)
Supplement: Supplementary file 1 [file ijms-24-04305-s001.zip › ijms-2169795-supplementary.pdf]

Supplementary materials

1.Supplementary methods

1.1 RNA-seq

RNA-seq was done by Shanghai Honsun Biological Technology Co., Ltd (China). Briefly, total RNA was isolated from tibias of ATF7ip$^{fl/fl}$ mice (n = 3) or Ocn-cre ATF7ip$^{fl/fl}$ mice (n = 3). RNA purity was tested by an Agilent 2100 Bioanalyzer (Agilent Technologies, CA, USA). Total RNA was used to construct a cDNA library. After purification by AMPure XP beads (Beckman Coulter, Beverly, USA), the library was quantified using Qubit2.0 Fluorometer and sequenced on Illumina NovaSeq 6000 (Illumina).

RNA-seq reads were aligned to the mouse genome (GRCm38/mm10) using HISAT2 2.0.5. Biological and technical replicates were used throughout. Transcripts were quantified using featureCounts (1.5.0-p3). Differential expression was analyzed using DESeq2 (1.20.0). Gene Ontology (GO) enrichment analysis of differentially expressed genes (DEGs), the statistical enrichment of DEGs in Kyoto Encyclopedia of Genes and Genomes (KEGG) pathways, and Reactome analyses for DEGs were accomplished on clusterProfiler R 3.8.1. Reactome pathways with corrected $P < 0.05$ were regarded significantly enriched by DEGs.

2.Supplementary figures and legends

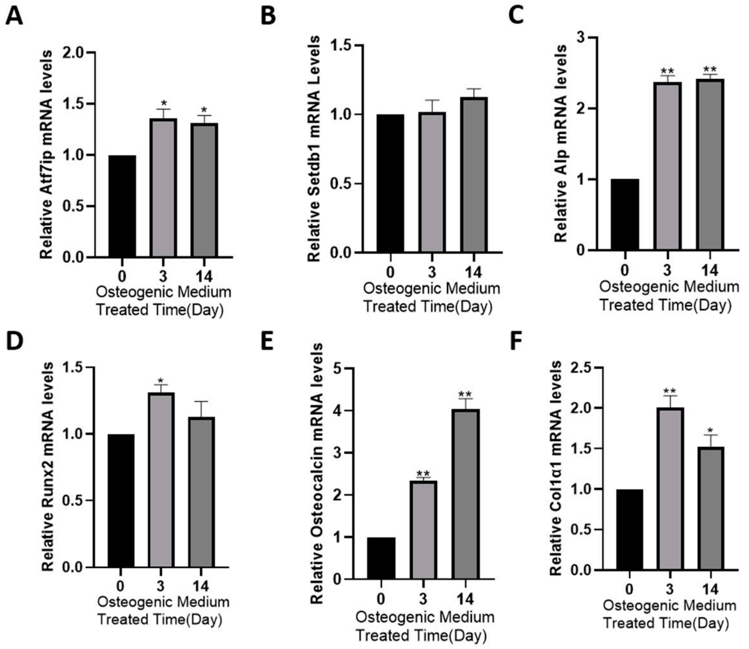

Figure S1. Atf7ip is upregulated during osteoblast differentiation. MC3T3-E1 cells were cultured in the osteoblast differentiation medium with osteogenic cocktail for the indicated days. The expressions of Atf7ip (A), Setdb1 (B) and osteoblast differentiation marker Alp (C), Runx2 (D), Osteocalcin (E) and Col1a1 (F) were detected by real-time PCR. Experiments were repeated at least three times.All the results were presented as mean ± SD (the same below). n=3-4, *p < 0.05, **p < 0.01, ***p < 0.001, treatments *vs* no treatment.

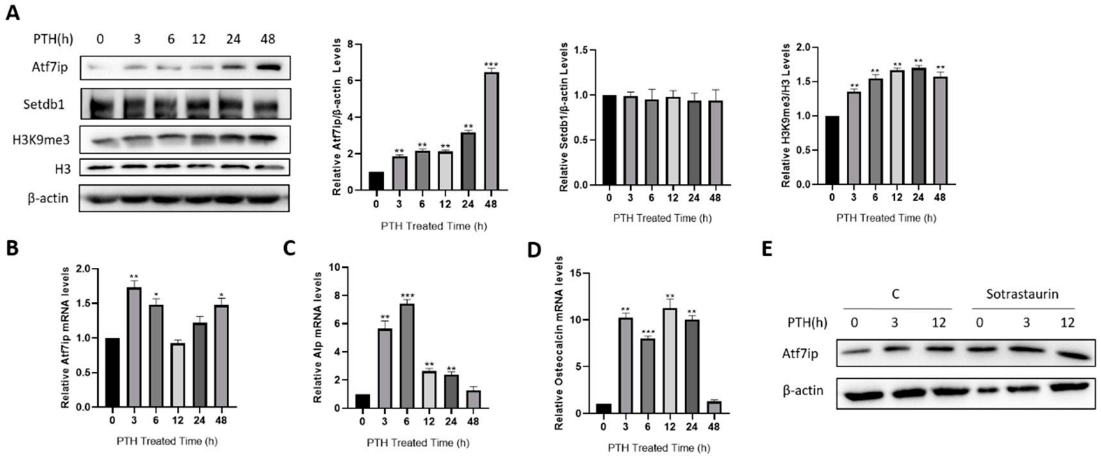

Figure S2. PTH induces Atf7ip expression in osteoblasts. C3H/10T1/2 cells were treated with PTH for different time. **(A)** protein expression and relative quantification of Atf7ip, Setdb1 and H3K9me3. **(B-D)** The mRNA expressions of Atf7ip (**B**), Alp (**C**) and Osteocalcin (**D**) examined by real-time PCR. (**E**) MC3T3-E1 cells were pretreated with or without Sotrastaurin (10 μM), followed by 500 ng/ml PTH treatment for 12 hours. Atf7ip protein level was detected by WB, with representative blots shown. β-actin was used as the loading control. Experiments were repeated at least three times. All the data in are presented as mean ±SD. n=3 *p < 0.05, **p < 0.01, ***p < 0.001; treatment *vs* no treatment.

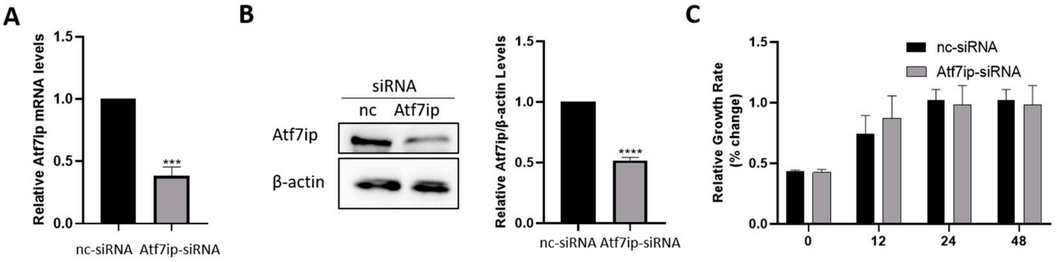

Figure S3. Atf7ip was knockdown with specific siRNA MC3T3-E1 cells received Atf7ip-siRNA treatment for 48 h, the effect of silencing Atf7ip was confirmed by real-time PCR **(A)** and Western blot(**B**). Cell growth rate was examined by CCK-8 analysis in Atf7ip-siRNA treated MC3T3-E1 cells (**C**). Atf7ip protein level was detected by WB, with representative blots shown. β-actin was used as the loading control. Experiments were repeated at least three times. All the data in are presented as mean ±SD. n=3 ***p < 0.001; ****p < 0.0001; Atf7ip-siRNA *vs* nc-siRNA.

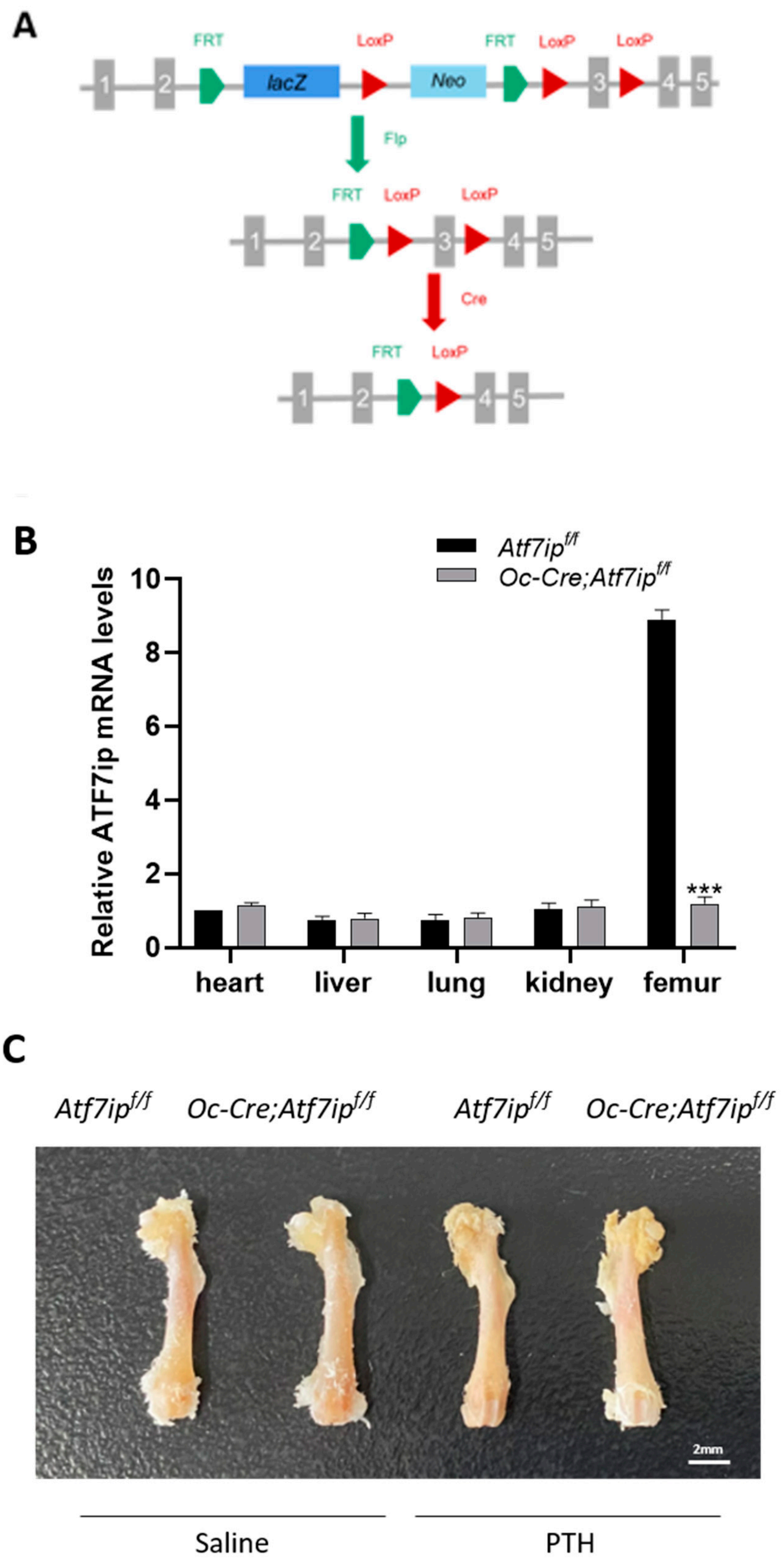

Figure S4. (A) Schematic of the 'knockout-first' conditional allele of *Atf7ip* mice. More detail can be found on previous paper. The 'knockout-first' allele has an IRES:lacZ trapping cassette and a

floxed promoter-driven neo cassette inserted into the intron of *Atf7ip*, disrupting gene function. Flp transforms the ‘knockout-first’ allele to a conditional allele. *Osteocalcin-Cre* deleted the floxed exon of the allele to form a frameshift mutation, initiating nonsense-modulated decay of the *Atf7ip* in mature osteoblast. **(B)** the mRNA for heart, liver, lung, and kidney of *Atf7ip*$^{Flox/Flox}$ mice (*Atf7ip*$^{f/f}$) and *Ocn-Cre;Atf7ip*$^{Flox/Flox}$ mice (*Oc-Cre;Atf7ip*$^{f/f}$) were extracted, and the specific knockout of *Atf7ip* in bone tissue was verified via qRT-PCR. Experiments were repeated at least three times. Values are mean ± SD. n=3, *p < 0.05, **p < 0.01, ***p < 0.001.

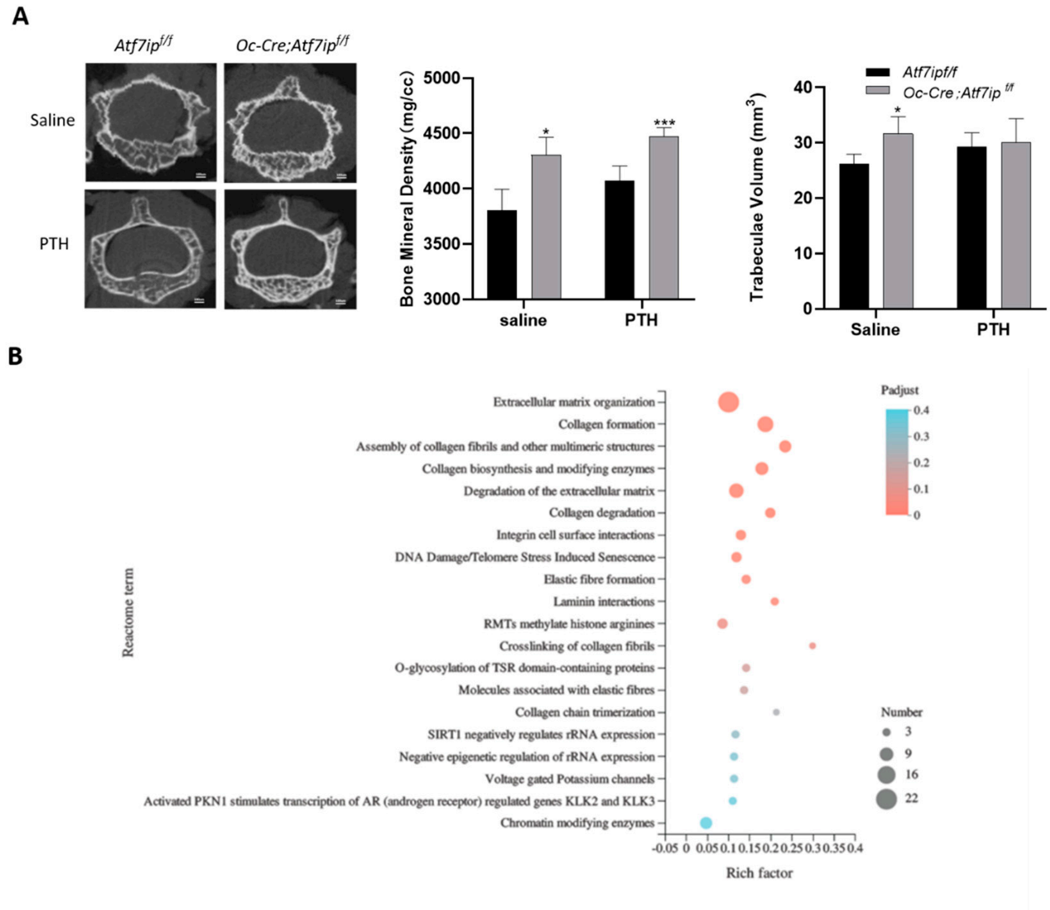

Figure S5. **(A)** Representative micro-CT images and calculated parameters from the vertebrae of male *Oc-Cre;Atf7ip*$^{f/f}$ (2 months old, n=6) and littermate control mice (*Atf7ip*$^{f/f}$) with or without PTH treatment. BMD, bone mineral density. **(B)** Reactome analyses for differential expression genes (*Oc-Cre;Atf7ip*$^{f/f}$ vs *Atf7ip*$^{f/f}$) were performed using clusterProfiler 3.8.1.
